# “The Mind Is Its Own Place”: Amelioration of Claustrophobia in Semantic Dementia

**DOI:** 10.1155/2014/584893

**Published:** 2014-03-06

**Authors:** Camilla N. Clark, Laura E. Downey, Hannah L. Golden, Phillip D. Fletcher, Rajith de Silva, Alberto Cifelli, Jason D. Warren

**Affiliations:** ^1^Dementia Research Centre, UCL Institute of Neurology, University College London, 8-11 Queen Square, London WC1N 3BG, UK; ^2^Essex Neurosciences Centre, Queen's Hospital, Rom Valley Way, Romford RM7 0AG, UK

## Abstract

Phobias are among the few intensely fearful experiences we regularly have in our everyday lives, yet the brain basis of phobic responses remains incompletely understood. Here we describe the case of a 71-year-old patient with a typical clinicoanatomical syndrome of semantic dementia led by selective (predominantly right-sided) temporal lobe atrophy, who showed striking amelioration of previously disabling claustrophobia following onset of her cognitive syndrome. We interpret our patient's newfound fearlessness as an interaction of damaged limbic and autonomic responsivity with loss of the cognitive meaning of previously threatening situations. This case has implications for our understanding of brain network disintegration in semantic dementia and the neurocognitive basis of phobias more generally.

## 1. Introduction

Specific phobia is defined in DSM-IVR as marked, persistent, and excessive or unreasonable fear when in the presence of, or when anticipating an encounter with, a specific object or situation [1]. Examples of specific phobias include animals (commonly mice, snakes, and spiders), natural environments (including heights, storms, or water), breaches of one's physical integrity (blood, injections and injury) and situations (notably, closed spaces or claustrophobia). Specific phobias are collectively common, with an estimated lifetime prevalence of around 10% in Western populations [1]. As rare instances of powerfully arousing, intensely fearful stimuli that are regularly encountered in modern developed societies, phobias hold potentially unique insights for our understanding of the cognitive and neural machinery of fear.

Functional imaging in human subjects suggests that specific phobias are neuroanatomically mediated by limbic and paralimbic circuitry including the amygdala, anterior cingulate, insula and dorsolateral prefrontal cortex, and subcortical connections to the ventral striatum and brainstem nuclei including locus coeruleus [2–6]. These brain regions are involved in the representation and interpretation of the phobic object, in amplification of the phobic response, and generation of the characteristic somatic correlates of extreme fear. Proximity of the phobic stimulus modulates activation in stria terminalis and orbitofrontal cortex, while mismatch between predicted and experienced fear engages the amygdala [2]. Supraliminally presented stimuli activate amygdala bilaterally whereas subliminally presented stimuli demonstrate lateralised activity in the right amygdala suggesting a role of the latter in hypervigilance to phobic stimuli before these attain conscious awareness [4]. The role of the amygdala is further underlined by the unique Urbach-Wiethe syndrome in which selective amygdalar proteinosis is accompanied by loss of fear responses [7]. Particular phobias vary in the extent to which they engage cognitive and autonomic components of the fear response [8, 9].

The frontotemporal lobar degenerations (FTLD) are a diverse group of proteinopathies that present clinically with impairments of social conduct and understanding, aphasias or deficits of conceptual knowledge about the world at large [10]. These diseases share a propensity to produce selective brain network disintegration maximally affecting the frontal and anterior temporal lobes [10]. Abnormal reactivity to and comprehension of a range of emotional stimuli are a hallmark of FTLD and in particular the canonical syndromic subtypes of behavioural variant frontotemporal dementia and semantic dementia (SemD). These deficits of emotion processing have been linked to regional atrophy and altered connectivity in frontolimbic circuitry, including orbitofrontal cortex, ventral striatum, insula, and amygdala [10–12]. The SemD syndrome is of particular interest because it is underpinned by selective erosion of semantic memory: the human memory system that governs conceptual and encyclopaedic knowledge about words and objects based on an individual's accumulated experience of the world. SemD is associated with progressive degeneration of a specific brain network centred on the anterior temporal lobes and their connections with inferior frontal, limbic, and more posterior brain regions [13]. SemD is most often led by loss of understanding of word meanings (progressive semantic aphasia) but less commonly can be led by deficits of nonverbal semantic memory, such as impaired face recognition (progressive associative prosopagnosia) [14]. Even in patients presenting with verbal semantic deficits, nonverbal semantic deficits are often detectable [15], and both verbal and nonverbal deficits progress as SemD unfolds, underlining the status of this syndrome as the paradigmatic disorder of the semantic memory system. It is increasingly recognised that SemD is associated with a range of behavioural disturbances that may be at least partly underpinned by severe deficits in comprehending affect-laden as well as affectively neutral objects and social concepts [12, 16, 17].

Here we describe the case of a patient in whom development of SemD was accompanied by striking attenuation of previously disabling claustrophobia, with implications both for our understanding of the pathophysiology of SemD and the brain basis of specific phobias.

## 2. General Clinical Details

This 71-year-old right-handed retired medical secretary, LC, presented with a seven-year history of cognitive decline led by progressive difficulty recognising familiar faces. More recently she had been unable to recognise even close friends and relatives and increasingly relied on other cues to their personal identity (e.g., the type of car they drove). She had also experienced difficulty recognising voices over the telephone. Word finding difficulties were an early feature and she struggled in particular to retrieve personal and brand names. Increasingly she seemed unable to understand how to use everyday household items or to comprehend environmental sounds. Her family had noted an insidious change in her personality and social behaviour beginning around three years after the onset of prosopagnosia and characterised by development of a sweet tooth, reduced empathy, loss of humour and social sensitivity, and increasing self-centredness, with obsessionality around time-keeping, picture puzzles, and music. There was no history of topographical disorientation. There was a past history of severe claustrophobia with previous psychiatric contact but no other significant past personal or family history.

**Figure 1:**
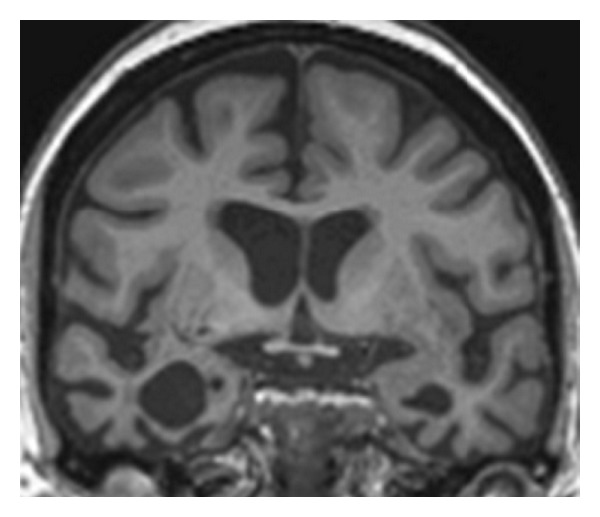
Representative coronal slice through the anterior temporal lobes from brain MRI in LC, six years after onset of symptoms (the right hemisphere is displayed on the left). There is selective atrophy of the anteroinferior and mesial temporal lobes including amygdalae and hippocampi (more marked on the right) and less marked atrophy of perisylvian cortices bilaterally.

Neuropsychological assessment (summarised in Table 1) corroborated the clinical impression: LC showed deficits of famous face recognition and visual object identification, anomia, and reduced single word comprehension, but her speech was fluent and normally constructed and there was relative preservation of her mnestic, perceptual and executive functions. The general neurological examination was unremarkable. Brain MRI (Figure 1) showed selective atrophy predominantly affecting the anteroinferior and mesial temporal lobes, more marked in the right hemisphere, with less marked atrophy of perisylvian cortices bilaterally. Based on LC's characteristic neuropsychological and neuroanatomical phenotype, a clinical diagnosis of SemD presenting with progressive prosopagnosia was made. This clinical diagnosis was additionally in line with current consensus criteria for the semantic variant of progressive aphasia, acknowledging that a minority of patients in this group do present with prominent difficulties with person recognition [13].

## 3. Alterations in Claustrophobia and Other Emotional Responses

A noteworthy feature of LC's history was striking attenuation of her previously disabling, longstanding claustrophobia following the onset of cognitive decline. She had been diagnosed by a psychiatrist with claustrophobia in her mid-twenties, and this had remained a significant issue throughout her adult life. Even in childhood, she had disliked being in crowded places such as the school chapel, and in her late teens and early twenties she exhibited mounting anxiety when in confined spaces including lifts, trains, aeroplanes, and other situations with no obvious route of escape. She would develop full-blown panic symptoms with sustained exposure to such situations and avoided them wherever possible, sometimes at the cost of considerable inconvenience (e.g., driving many kilometres out of her way to avoid road tunnels or planning vacations around her fear). There had been no suggestion of a generalised anxiety disorder nor any history of other phobic responses. Her family reported that LC's claustrophobia settled within several years of onset of her cognitive symptoms: she would, for example, now travel willingly on the London Underground and enter crowded lifts when accessing the platforms. A compelling illustration occurred some six years following symptom onset, when she agreed to have a brain MRI and underwent the procedure with no evidence of distress. Indeed, her family remarked that loss of her claustrophobia was the one positive outcome of LC's SemD diagnosis.

On specific enquiry, there was the suggestion of a more general alteration in LC's emotional responses. In earlier life she had been prone to fairly regular vociferous, angry outbursts; these had abated following the onset of cognitive decline. In addition, she now failed to react to situations likely to have provoked disgust premorbidly (e.g., leaving her washing machine filled with stagnant water and accumulating cartons of mouldy food in her house). In contrast to this reduction in certain strong premorbid emotional responses, LC had developed a craving for music (musicophilia), repeatedly requesting to hear the same repertoire of songs derived from Hollywood musicals. She evidently derived considerable pleasure from these songs and, before her family intervened, would stay up late into the night listening to them.

## 4. Discussion

This patient with SemD lost phobic responses to a specific situation (confined spaces) that had previously reliably evoked them, following the onset of her cognitive syndrome. Although this amelioration of claustrophobia probably occurred in the context of a more generalised alteration of strong emotional responsivity, it is nevertheless a striking illustration of the specific modulation of an established behavioural programme by neurodegenerative disease. This case has implications both for our understanding of brain network disintegration in SemD and the neurocognitive basis of phobias more generally.

From a disease perspective, this case shows that the well-recognised effects of SemD on emotion processing extend even to essentially “automatic” and powerful emotional behaviours (such as phobic responses) that have become highly entrenched over the course of a lifetime. While gratifying in this particular patient, there is clear potential for harm here: whereas phobias* per se* are a nonuseful legacy of our evolutionary past, the capacity to experience fear when confronted with genuinely threatening scenarios remains highly useful. If SemD is accompanied by a more generalised fearlessness, this could leave patients vulnerable in their daily lives: however, there remain few data on the real life impact of altered emotion processing in patients with neurodegenerative diseases [12, 16, 28, 29]. Pathophysiologically, while direct neuroanatomical correlation was not possible here, the pattern of regional brain atrophy exhibited by LC (see Figure 1) suggests that her loss of phobic responses is attributable to involvement of key structures such as the amygdala, insula, and their connections. Aversive learning paradigms in neurodegenerative dementia cohorts have shown that FTLD (in contrast to Alzheimer's disease) is associated both with blunted fear conditioning and reduced autonomic responsivity, and this is in turn correlated with grey matter loss in a large-scale emotion processing network including anterior cingulate, orbitofrontal cortex, and insula [30]. The development of musicophilia in LC's case suggests that SemD does not simply produce a global attenuation of emotionality: rather the specific stimulus may modulate the valence of the abnormal emotional response, presumably owing to stimulus-dependent alterations of connectivity in cognitive and limbic circuitry, as proposed in previous cases of musicophilia associated with SemD [31].

From a neurocognitive perspective, the present case particularly implicates the right temporal lobe in the modulation of phobic responses (since loss of claustrophobia in this case evolved in tandem with prosopagnosia and predominantly right-sided anterior temporal lobe atrophy) and raises the further possibility that the phenomenology of a phobia reflects conjoint processing of both the experience of strong emotion [32] and the cognitive “meaning” of the experience. Moral sentiments experienced by healthy individuals have been shown to be mediated through emotion-specific functional connectivity between the anterior temporal lobe and frontolimbic regions, providing a potential mechanism for conceptual-emotional integration [33]. Disease-associated neuronal dysfunction and loss in SemD affect both temporal and frontal lobe neocortex as well as the subcortical machinery of autonomic responses. We speculate that neocortical processing in the otherwise healthy brain underpins the more complex aspects of phobic behaviour (such as LC's elaborate premorbid avoidance strategies), while impaired neocortical processing (e.g., following onset of SemD) may remove the cognitive significance of the phobic situation along with its autonomic resonance. It may be that attenuation of both these components of the phobic experience is required to “cure” the phobia, as indeed psychologically based treatment strategies would suggest [34, 35].

Conclusions based on single case studies must necessarily be tentative. However we hope that our observations in this case will motivate a further systematic neuropsychological and psychophysical investigation of phobic (and potentially phobic) responses in patients with FTLD, in relation to other neurodegenerative diseases and with electrophysiological and neuroimaging correlation. More philosophically, our case illustrates how the brain constructs a private model of the world and invests this with emotional significance and how this process can be modulated by pathological mental states, as recognised long ago by Milton [36] and others.

## Figures and Tables

**Table 1:** Neuropsychological profile of LC 6 years after onset of symptoms.

Cognitive domain	Raw score	Percentile*/normal range^†^
*General intellect (WASI) *		
Verbal IQ	84	
Performance IQ	93	

*Executive function *		
Stroop test: colour-word inhibition	60 s	25–50th
WMS-R Digit Span:		
Forwards	9/12 Max: 7	50th–75th
Backwards	9/12 Max: 6	90th–95th

*Episodic memory *		
Visual recognition:		
Faces	39/50	10th
Words	27/50	5th

*Language *		
Graded Naming Test	0/30	<1st
Synonyms:		
Concrete	18/25	<2nd
Abstract	16/25	<2nd
British Picture Vocabulary Scale (BPVS)	136/150	>144/150^†^
Pyramids and palm trees—pictures	45/52	<5th
Reading (NART)	27/50	N/A

*Semantic memory: faces *		
Famous faces: recognition	7/12	<10th^‡^
Famous faces: naming	2/12	<5th^†^

*Visual perceptual *		
Object decision VOSP	16	5–25th
Incomplete letters	20/20	>99th
Position discrimination	20/20	>99th

*As applicable using WASI: Wechsler Abbreviated Scale of Intelligence [18]; Stroop, Delis-Kaplan Executive Function System Stroop Test [19]; Recognition Memory Tests [20]; GNT: Graded Naming Test [21]; Concrete and Abstract Word Synonym Test [22]; BPVS: British Picture Vocabulary Scale [23]; Pyramids and Palm Trees Test [24]; NART: National Adult Reading Test [25]; VOSP: Visual Object and Space Perception Battery [26].

^†^Based on normative data from an historical group of 100 healthy controls aged 55–70 years [27].

^‡^Local unpublished normative data from 310 controls aged 55–70 years.
